# Development of a predictive model for in-hospital new-onset atrial fibrillation in older adults with hypertension and acute myocardial infarction, enhanced by SHAP interpretability: a retrospective cohort study

**DOI:** 10.3389/fmed.2026.1747281

**Published:** 2026-03-06

**Authors:** Xue Ge, Yang Tao, Lin Zhang, Jiali Cao, Xingmei Dong, Lixiang Ma

**Affiliations:** 1 Department of Ultrasound, The First Hospital of Qinhuangdao, Qinhuangdao, Hebei, China; 2 Central Laboratory, The First Hospital of Qinhuangdao, Qinhuangdao, Hebei, China; 3 Department of Hematology, The First Hospital of Qinhuangdao, Qinhuangdao, Hebei, China; 4 Department of Cardiology, The First Hospital of Qinhuangdao, Qinhuangdao, Hebei, China

**Keywords:** acute myocardial infarction, new-onset atrial fibrillation, nomogram, predictive model, SHAP value

## Abstract

**Background:**

Acute myocardial infarction (AMI) remains a leading cause of mortality, particularly among older adults with hypertension, who are at a heightened risk for complications such as new-onset atrial fibrillation (NOAF). Despite existing research, predictive models for NOAF in this population are limited in both scope and clinical utility, often lacking interpretability, which hinders their use in clinical practice.

**Objective:**

This study aims to develop and validate a predictive model for NOAF in older adults with hypertension who have experienced AMI, incorporating machine learning techniques and SHapley Additive exPlanations (SHAP) value to enhance the model’s interpretability and clinical utility.

**Methods:**

A retrospective cohort study was conducted on 2,140 older hypertensive adults hospitalized with AMI at the First Hospital of Qinhuangdao. Key features were selected using Boruta, LASSO regression, and logistic regression. A predictive nomogram was constructed via multivariate logistic regression, and SHAP value was utilized to explain the model’s predictions. The model’s performance was assessed using ROC, AUC, calibration curves, and clinical utility was evaluated via Decision Curve Analysis and Clinical Impact Curves.

**Results:**

The model identified eight key predictors: age, left atrial diameter, ejection fraction, white blood cell count, triglycerides, low-density lipoprotein, NT-proBNP, and potassium. The nomogram demonstrated excellent discrimination with an AUC of 0.895 in the training set and 0.883 in the validation set. An interactive web-based tool was developed (https://longmao.shinyapps.io/NOAF/) to provide real-time NOAF risk predictions. SHAP values clarified feature contributions, enhancing the model’s interpretability and clinical relevance.

**Conclusion:**

This study presents an interpretable predictive model for NOAF in older adults with hypertension and AMI, introduces an online tool for real-time exploratory risk assessment. The model demonstrates high discriminative performance and potential clinical relevance for early detection and personalized management of high-risk patients.

## Introduction

Acute Myocardial Infarction is one of the leading cardiovascular diseases with a high mortality rate globally (1). Although advancements in modern medicine have improved the treatment outcomes and survival rates for AMI, its high mortality during the acute phase, as well as complications such as heart failure, arrhythmias, and left ventricular thrombus, continue to make it a significant challenge in global healthcare management (2). The occurrence of AMI is closely related to atherosclerosis, coronary artery stenosis, and thrombosis, all of which lead to inadequate blood supply to the heart, resulting in myocardial injury or necrosis.

Among older adults with hypertension, the incidence of AMI and the risk of complications are significantly elevated. Hypertension is a major risk factor for coronary artery atherosclerosis, as the long-term effects of high blood pressure cause thickening and stiffening of the arterial walls, contributing to coronary artery narrowing and occlusion (3, 4). As individuals age, the cardiovascular function of elderly hypertensive patients progressively declines, and the long-term impact of hypertension on the structure and function of the heart places these patients at a higher risk for AMI. Research indicates that older adults with hypertension experience higher mortality and complication rates following AMI compared to the general population, with a greater incidence of complications such as heart failure and recurrent infarction (5).

NOAF is a common complication in patients with AMI, particularly among older adults with coexisting hypertension, where the occurrence of NOAF significantly increases the risk of mortality and long-term complications (6, 7). Older adults with hypertension and AMI are more prone to atrial fibrillation due to structural changes in the heart, such as left atrial enlargement and left ventricular hypertrophy. Hypertension exacerbates the load on the left atrium, accelerating its enlargement and creating favorable conditions for the onset of atrial fibrillation (8, 9). Additionally, the atherosclerosis associated with hypertension leads to more unstable coronary blood flow, increasing the risk of cardiac electrophysiological abnormalities, which further promote the development of atrial fibrillation (10). Therefore, the occurrence of NOAF in this patient group not only increases the cardiac burden but also complicates their management. These patients require special attention to anticoagulation therapy and other cardiovascular management strategies to reduce the risk of embolic events, such as stroke, while also optimizing strategies for heart failure management.

Although numerous studies have explored the risk factors for NOAF, such as age, left atrial size, and cardiac function parameters, existing prediction models are often based on single or partial clinical variables (11). These models fail to comprehensively account for the various factors influencing the occurrence of NOAF in older adults with hypertension who experience AMI, and therefore, a simple and effective predictive tool is lacking. Consequently, there is an urgent need to develop a prediction model for new-onset atrial fibrillation in this specific population to enable early identification of high-risk patients and timely intervention.

In recent years, machine learning methods have made significant strides in the medical field, particularly in disease prediction and risk assessment. However, machine learning models are often regarded as “black boxes,” with poor interpretability, limiting their widespread application in clinical practice (12). To address this issue, the SHAP method has emerged. Based on the Shapley values from game theory, SHAP assigns an importance value to each feature, helping to explain the model’s predictions. Research has shown that the application of SHAP in the medical field improves the transparency of models and enhances clinicians’ trust in these models (13). Thus, this study aims to develop an online web-based prediction model for NOAF in older adults with hypertension hospitalized for AMI, in which machine learning–assisted feature selection and SHAP-based interpretability are integrated to support clinical risk assessment. Through a retrospective analysis of clinical data from elderly hypertensive AMI patients, a multivariable logistic regression–based nomogram will be constructed, with machine learning–assisted feature screening used to identify high-risk factors for NOAF, and SHAP employed to explain the contributions of individual variables to model predictions. This study will provide clinicians with an effective tool to help early identification of high-risk patients and the development of personalized treatment plans, ultimately improving patients’ long-term prognosis.

## Method

### Study participants

This was a retrospective study conducted on hospitalized patients admitted to the Department of Cardiology at the First Hospital of Qinhuangdao from July 2019 to December 2024. The inclusion criteria were as follows: (1) patients aged 65 years and older; (2) diagnosis of AMI according to the criteria set by the European Society of Cardiology; and (3) availability of complete medical records, laboratory results, and other necessary clinical documentation. Patients meeting any of the following criteria were excluded: (1) absence of hypertension; (2) history of atrial fibrillation (AF) upon admission, including secondary persistent AF or paroxysmal AF; (3) patients who did not undergo coronary angiography; and (4) incomplete medical records, laboratory findings, echocardiographic data, or other essential clinical data.

### Ethical statement

This study was a retrospective analysis based on existing patient medical records. All patient data collection and analysis procedures were anonymized to ensure patient confidentiality and privacy protection. Additionally, the study protocol was approved and supported by the Institutional Review Board of the First Hospital of Qinhuangdao and the Qinhuangdao S&T Plan Program (No. 202501A129).

### Definition of disease

AMI is an acute ischemic event of the myocardium, typically caused by a sudden obstruction of coronary blood flow, leading to inadequate supply to meet myocardial demand, resulting in an imbalance between supply and demand. According to the latest guidelines from the ESC and the AHA/ACC, the diagnosis of AMI relies on clinical symptoms (such as typical chest pain or discomfort, potentially accompanied by nausea, vomiting, sweating, or shortness of breath), the dynamic evolution of the electrocardiogram (including ST-segment elevation or new-onset left bundle branch block, as well as possible ST-segment depression or T-wave inversion), elevated myocardial injury biomarkers, such as troponins T and I, or myocardial band isoenzyme (CK-MB) levels greater than twice the upper limit of normal, and imaging evidence showing new myocardial injury or dysfunction (14, 15).

NOAF is defined as the first occurrence of atrial fibrillation in a patient with no previous history of AF. The diagnosis of NOAF is based on cardiac monitoring data obtained during hospitalization, commonly confirmed through Holter monitoring, 12-lead electrocardiogram, or continuous electrocardiographic monitoring. Electrocardiogram features include the absence of P-waves, irregular R-R intervals, and an irregular rhythm, with a duration lasting more than 30 s. NOAF typically presents as paroxysmal atrial fibrillation, meaning the episodes occur suddenly, usually lasting no more than 48 h, and may terminate spontaneously. During diagnosis, confirmation should be made through a comprehensive analysis of electrocardiogram characteristics, clinical symptoms, and the patient’s medical history (16). In routine clinical practice, the type and duration of cardiac rhythm monitoring were determined by standard clinical care rather than a uniform study-specific protocol.

### Data collection

Relevant data were collected from the hospital’s medical record system after patient admission. The basic data collected included: age, body mass index (BMI), gender, smoking status, alcohol consumption, heart rate at admission, systolic blood pressure at admission, and diastolic blood pressure at admission. The medical history primarily included coronary artery disease, previous cerebral infarction, chronic renal failure, diabetes, chronic obstructive pulmonary disease, cancer, and valvular heart disease. In-hospital complications primarily included pulmonary infections, ventricular arrhythmias, acute kidney injury, stress ulcers, and other laboratory and examination data such as white blood cell count (WBC), hemoglobin, platelet count, total cholesterol, triglycerides, high-density lipoprotein (HDL), low-density lipoprotein (LDL), glycated hemoglobin (HbA1c), random blood glucose, high-sensitivity C-reactive protein, cardiac troponin I, NT-proBNP, potassium, sodium, and albumin levels. Echocardiographic data included left ventricular end-diastolic diameter (LVEDD), left atrial diameter (LADD), ejection fraction (EF), and cardiac output (CO). Additional data on AMI type, Killip classification, and coronary angiography results were also collected.

### Variable selection and model construction

In this study, all statistical analyses were conducted using SPSS 24.0 and R software. The statistical significance level was set at *p* < 0.05. The research strictly adhered to the TRIPOD guidelines for the transparent development and reporting of predictive models. First, the collected data were divided into training and validation sets following a 70:30 split, and descriptive statistical analysis was performed on the baseline characteristics of the participants. All subsequent feature selection procedures were performed exclusively within the training set to avoid information leakage. Next, the Least Absolute Shrinkage and Selection Operator (LASSO) method, Brouta, and univariate logistic regression analysis were applied in parallel to the training set data to identify risk factors significantly associated with NOAF. The identified risk factors were then intersected, and the relationships between the selected variables were displayed and analyzed using UpSet plots and network graphs. This complementary selection strategy was adopted to balance statistical significance, model stability, and the ability to capture potential non-linear relationships. Based on this, a multivariate logistic regression model was further used to assess the relationship between these factors and NOAF, and a forest plot was constructed to visually represent the findings. This approach ensured that only statistically significant predictors were included in the final model. The Variance Inflation Factor (VIF) and tolerance were calculated to assess potential collinearity between parameters. A VIF value below 5 and a tolerance above 0.1 were considered indicative of no significant collinearity. To provide a more intuitive representation of these collinearity issues, VIF plots and Pearson correlation matrix plots were used to further analyze the correlations between variables, ensuring that only statistically significant factors remained in the final model. Additionally, nomograms and an online web-based evaluation tool were developed to visually present the model’s predictions and the contribution of each variable, making it easier for clinicians to perform online assessments.

### Model evaluation and interpretation

In the model evaluation phase, we first assessed the model’s discriminatory ability by plotting the Receiver Operating Characteristic (ROC) curve and calculating the Area Under the Curve (AUC) value. Next, we used the Hosmer-Lemeshow test to evaluate the model’s calibration. To further assess the model’s practical application in clinical decision-making, we employed Decision Curve Analysis (DCA). DCA helps calculate the net benefit at different thresholds and provides a comprehensive evaluation of the model’s clinical decision value. To further investigate the model’s impact in a real-world clinical setting, we introduced Clinical Impact Curves (CIC), which were visualized at different threshold settings to show how the model’s predictions influence clinical interventions (such as the decision to treat). CIC aids in optimizing the model’s clinical application, ensuring it provides the maximum health benefits while minimizing unnecessary medical interventions.

Additionally, to enhance the model’s interpretability and particularly to increase clinicians’ confidence in the model’s predictions, we incorporated the SHAP method. SHAP, based on Shapley values from game theory, assigns a weight to each feature, thus helping explain the model’s predictions. In this study, SHAP was used to explain the impact of each variable in the model on predicting NOAF. Through SHAP’s visualizations, we can clearly see the importance and weight distribution of each clinical variable in the model, helping clinicians understand which factors contribute most to the prediction of NOAF, and thus support the development of personalized treatment plans.

## Results

### Baseline characteristics of patients

**Figure 1:**
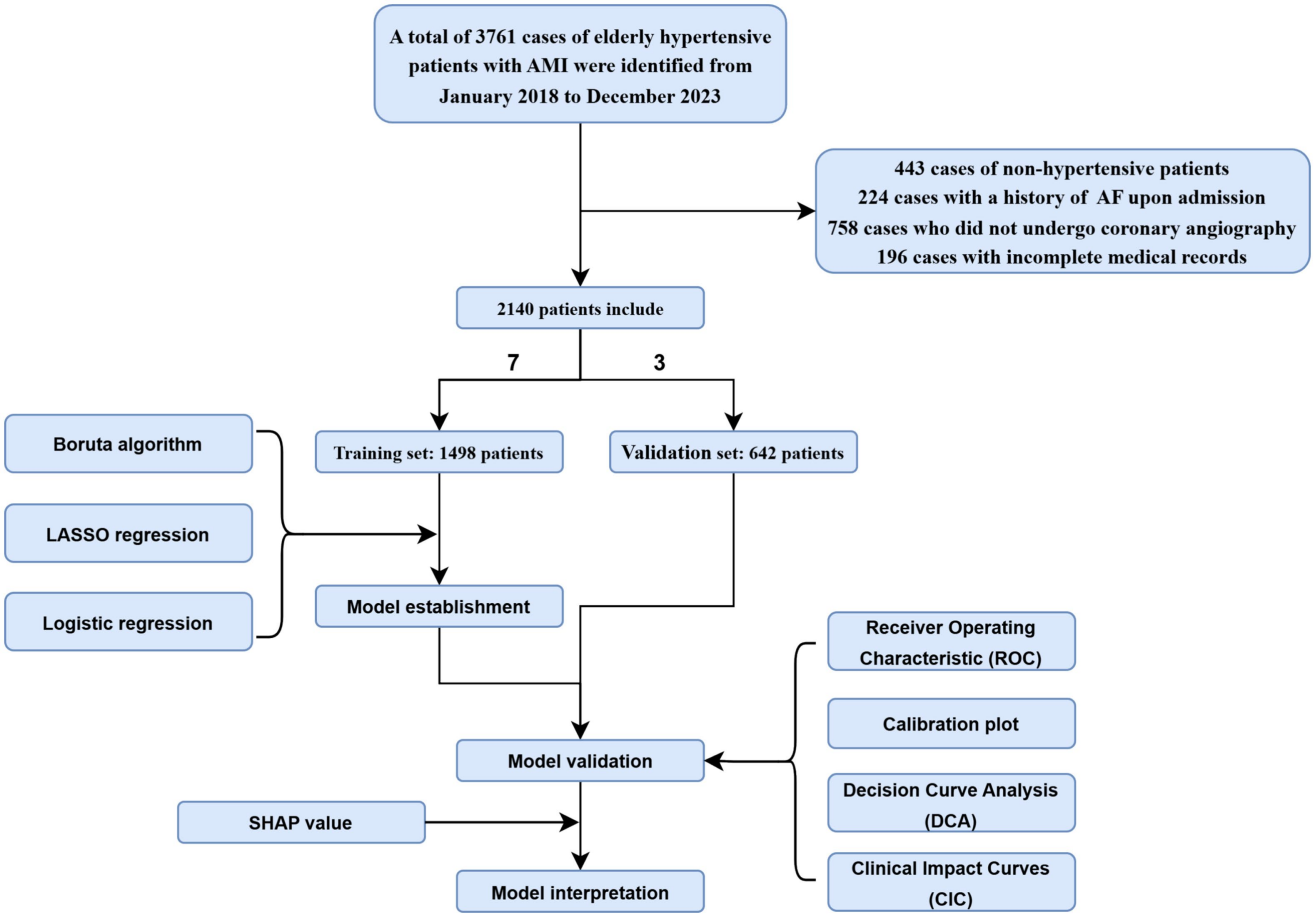
The flow chart in our study. The dataset was partitioned into training set and validation set in a 7:3 ratio. Feature selection was performed using Boruta, LASSO, and logistic regression. A predictive model was developed and validated through ROC curves, calibration plots, DCA, and CIC. SHAP values were applied to enhance model interpretability.

Between January 2018 and December 2023, a total of 3,761 older adults diagnosed with AMI were included in the study. After screening, 1,621 patients were excluded, leaving 2,140 patients for final inclusion. The excluded patients included 443 non-hypertensive patients, 224 patients with a history of AF at admission, 758 patients who did not undergo coronary angiography, and 196 patients with incomplete data. The included patients were randomly divided into training and validation sets using a 70:30 ratio, with 1,498 patients assigned to the training set and 642 patients to the validation set (Figure 1). We compared the baseline characteristics of both groups, and the results showed no significant differences in most clinical and laboratory parameters between the training and validation sets (*p* > 0.05), indicating that the two groups were statistically comparable and suitable for subsequent model training and validation (Table 1). Baseline characteristics of patients with and without NOAF in the training cohort are summarized in Supplementary Table S1.

**Table 1:** Baseline clinical characteristics in training group and validation group.

Variables	Total (*N* = 2,140)	Training group (*N* = 1,498)	Validation group (*N* = 642)	*p*-value
Atrial fibrillation, *N* (%)				0.840
No	1,721 (80.4%)	1,203 (80.3%)	518 (80.7%)	
Yes	419 (19.6%)	295 (19.7%)	124 (19.3%)	
Gender, *N* (%)				0.962
Male	1,165 (54.4%)	816 (54.5%)	349 (54.4%)	
Female	975 (45.6%)	682 (45.5%)	293 (45.6%)	
Age, mean ± SD (years)	73.83 ± 5.96	73.82 ± 5.99	73.87 ± 5.89	0.866
BMI, mean ± SD	24.52 ± 2.28	24.54 ± 2.29	24.48 ± 2.25	0.603
Heart rate at admission, mean ± SD	84.42 ± 15.45	84.38 ± 15.27	84.51 ± 15.86	0.861
Systolic blood pressure at admission, mean ± SD	147.54 ± 21.81	147.48 ± 21.85	147.69 ± 21.72	0.839
Diastolic blood pressure at admission, mean ± SD	79.68 ± 12.34	79.68 ± 12.48	79.67 ± 12.01	0.979
Smoking history, *N* (%)				0.614
No	1,393 (65.1%)	970 (64.7%)	423 (65.9%)	
Yes	747 (34.9%)	528 (35.3%)	219 (34.1%)	
Drinking history, *N* (%)				0.019
No	1,796 (83.9%)	1,239 (82.7%)	557 (86.8%)	
Yes	344 (16.1%)	259 (17.3%)	85 (13.2%)	
Comorbidity, *N* (%)				
Coronary heart disease				0.529
No	1,540 (72.0%)	1,072 (71.6%)	468 (72.9%)	
Yes	600 (28.0%)	426 (28.4%)	174 (27.1%)	
Chronic renal failure				0.118
No	2,094 (97.8%)	1,461 (97.5%)	633 (98.6%)	
Yes	46 (2.2%)	37 (2.5%)	9 (1.4%)	
Diabetes				0.007
No	1,732 (80.9%)	1,235 (82.4%)	497 (77.4%)	
Yes	408 (19.1%)	263 (17.6%)	145 (22.6%)	
Heart valve disease				0.756
No	1,920 (89.7%)	1,342 (89.6%)	578 (90.0%)	
Yes	220 (10.3%)	156 (10.4%)	64 (10.0%)	
Old cerebral infarction				0.651
No	1,693 (79.1%)	1,189 (79.4%)	504 (78.5%)	
Yes	447 (20.9%)	309 (20.6%)	138 (21.5%)	
Cancer				0.802
No	2,077 (97.1%)	1,453 (94.0%)	624 (97.2%)	
Yes	63 (2.9%)	45 (3.0%)	18 (2.8%)	
COPD				0.587
No	1,874 (87.6%)	1,308 (87.3%)	566 (88.2%)	
Yes	266 (12.4%)	190 (12.7%)	76 (11.8%)	
Complications, *N* (%)				
Pulmonary infection				0.309
No	1,866 (87.2%)	1,299 (86.7%)	567 (88.3%)	
Yes	274 (12.8%)	199 (13.3%)	75 (11.7%)	
Ventricular arrhythmia				0.230
No	1,817 (84.9%)	1,281 (85.5%)	536 (83.5%)	
Yes	323 (15.1%)	217 (14.5%)	106 (16.5%)	
Acute kidney injury				0.601
No	1,970 (92.1%)	1,382 (92.3%)	588 (91.6%)	
Yes	170 (7.9%)	116 (7.7%)	54 (8.4%)	
Stress ulcer				0.331
No	2,135 (99.8%)	1,493 (99.7%)	642 (100.0%)	
Yes	5 (0.2%)	5 (0.3%)	0 (0.0%)	
Echocardiogram results, mean ± SD				
LVEDD	55.95 ± 5.74	55.86 ± 5.70	56.16 ± 5.83	0.269
LADD	37.21 ± 3.90	37.17 ± 3.96	37.30 ± 3.79	0.467
EF	51.28 ± 7.99	51.34 ± 8.15	51.13 ± 7.61	0.566
CO	6.07 ± 2.07	6.04 ± 2.08	6.13 ± 2.04	0.344
Laboratory data, mean ± SD				
WBC	9.95 ± 1.17	9.96 ± 1.16	9.92 ± 1.20	0.416
Hemoglobin	129.12 ± 17.96	129.34 ± 17.91	128.59 ± 18.06	0.372
PLT	178.28 ± 58.45	178.57 ± 58.53	177.60 ± 58.24	0.725
Total cholesterol	5.08 ± 1.39	5.09 ± 1.39	5.06 ± 1.37	0.643
Triglycerides	1.55 ± 0.35	1.55 ± 0.34	1.55 ± 0.35	0.975
High density lipoprotein	1.16 ± 0.18	1.16 ± 0.18	1.15 ± 0.18	0.202
Low density lipoprotein	2.98 ± 0.66	2.97 ± 0.67	2.98 ± 0.63	0.811
Glycosylated hemoglobin	5.75 ± 1.22	5.78 ± 1.21	5.70 ± 1.23	0.195
Random Blood Sugar	7.75 ± 2.92	7.75 ± 2.90	7.73 ± 2.97	0.875
hsCRP	5.18 ± 2.82	5.21 ± 2.86	5.10 ± 2.73	0.385
cTNI	3.44 ± 1.75	3.43 ± 1.75	3.48 ± 1.75	0.566
NT-proBNP	1170.59 ± 217.59	1169.17 ± 217.14	1173.91 ± 218.59	0.644
Potassium	3.89 ± 0.50	3.89 ± 0.50	3.89 ± 0.49	0.818
Sodium	141.96 ± 5.07	141.95 ± 5.10	141.99 ± 5.00	0.857
Albumin	40.14 ± 6.60	40.06 ± 6.55	40.33 ± 6.72	0.390
Type of AMI, *N* (%)				0.299
STEMI	1,060 (49.5%)	731 (48.8%)	329 (51.2%)	
NSTEMI	1,080 (50.5%)	767 (51.2%)	313 (48.8%)	
Killip class, *N* (%)				
Killip ≤ 2	1,748 (81.7%)	1,232 (82.2%)	516 (80.4%)	0.306
Killip > 2	392 (18.3%)	266 (17.8%)	126 (19.6%)	
Culprit lesion				
LM, *N* (%)	46 (2.15%)	36 (2.40%)	10 (1.56%)	0.216
LAD, *N* (%)	1,487 (69.47%)	1,042 (69.56%)	445 (69.32%)	0.910
LCX, *N* (%)	1,599 (74.72%)	1,122 (74.90%)	477 (74.30%)	0.769
RCA, *N* (%)	1,333 (62.29%)	930 (62.08%)	403 (62.77%)	0.763

Values are presented as mean ± standard deviation, median (interquartile range), or number (percentage) as appropriate. SD, standard deviation; BMI, Body mass index; COPD, chronic obstructive pulmonary disease; EF, ejection fraction; LVEDD, left ventricular end-diastolic volume; LADD, left atrial diameter; CO, cardiac output; WBC, white blood cell count; PLT, platelet count; CRP, C-reactive protein; LM, left main coronary artery; LAD, left anterior descending artery; LCX, left circumflex artery; RCA, right coronary artery.

### Feature selection

**Figure 2:**
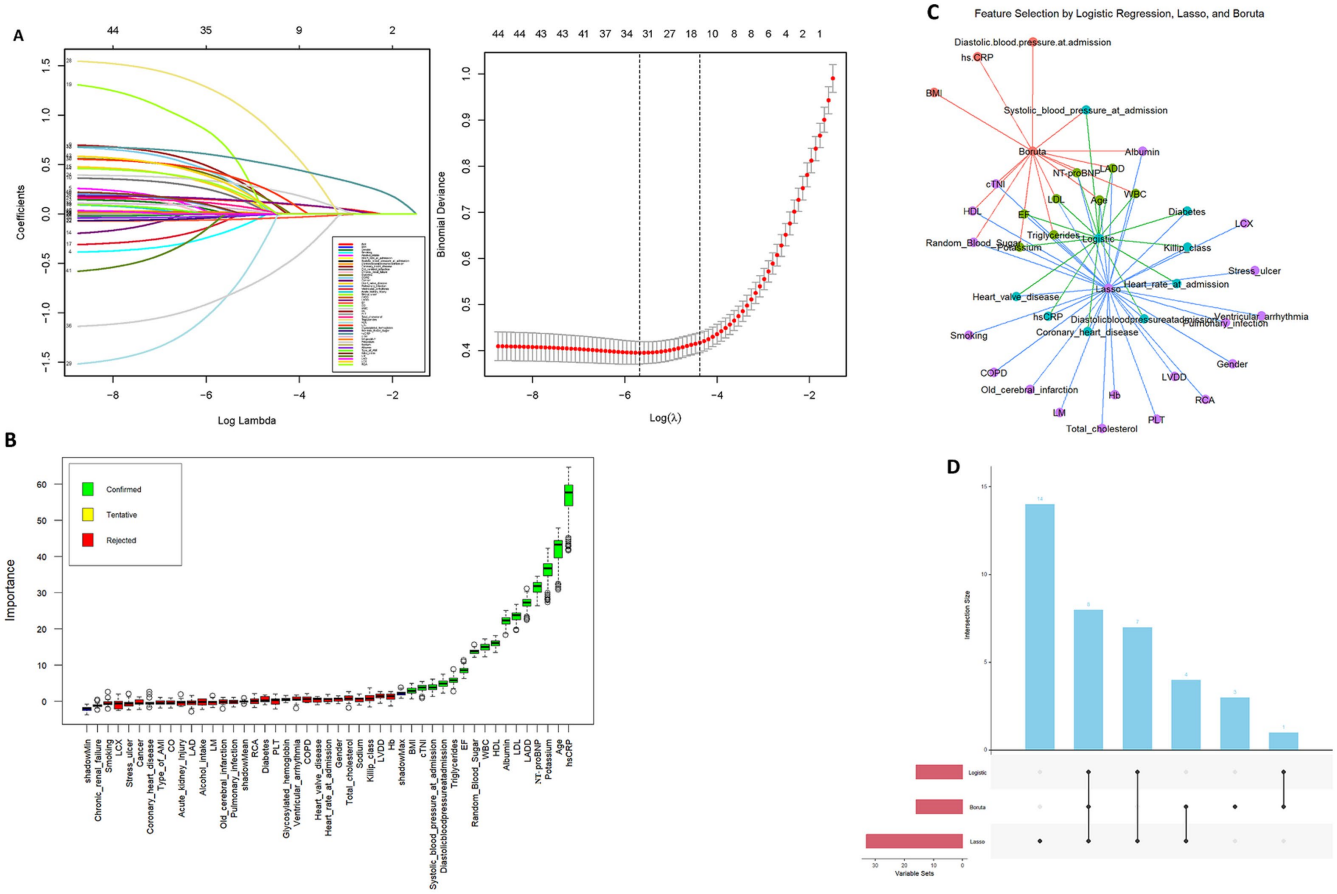
Feature selection using LASSO, Boruta, and logistic regression methods. **(A)** LASSO regression for variable selection: the left panel shows coefficient profiles with increasing penalty (log λ), and the right panel identifies the optimal λ value based on minimum binomial deviance. **(B)** Boruta feature selection results: green indicates confirmed important variables, yellow for tentative, and red for rejected ones. **(C)** Network graph showing intersected features selected by LASSO, logistic regression, and Boruta, highlighting core predictors such as NT-proBNP, EF, potassium, and LADD. **(D)** UpSet plot displaying the intersection of variables across the three selection methods, with eight key features identified by all.

In this study, we employed three complementary approaches for feature screening on the training set data, including machine learning–assisted methods (Boruta algorithm and LASSO regression) and univariate logistic regression analysis. First, the Boruta algorithm was used for feature selection. Boruta, a random forest–based feature selection algorithm, identifies relevant features by comparing the importance of each feature with that of its randomly generated “shadow feature.” Using this method, Boruta identified 16 variables that were closely related to the risk of NOAF. Next, we applied LASSO regression to process the data. LASSO, through L1 regularization, performs feature selection by eliminating redundant features and retaining the most predictive variables. The results from LASSO regression showed that 34 variables exhibited strong correlation with the prediction of NOAF. We then used univariate Logistic regression for further feature selection, and the results indicated that 16 variables were significantly associated with the occurrence of NOAF. To further analyze the intersection of these features, we visualized the features selected by the three methods using UpSet plots and network graphs, clearly showing the overlap between the variables selected by each method (Figure 2). Through this intersection analysis, we identified 8 variables that were selected in all three methods (Boruta, LASSO regression, and Logistic regression), which include: Age, LADD, EF, WBC, Triglycerides, LDL, NT-proBNP, and Potassium. These overlapping variables suggest that they may play a core role in the prediction model for NOAF.

### Model construction and validation

**Figure 3:**
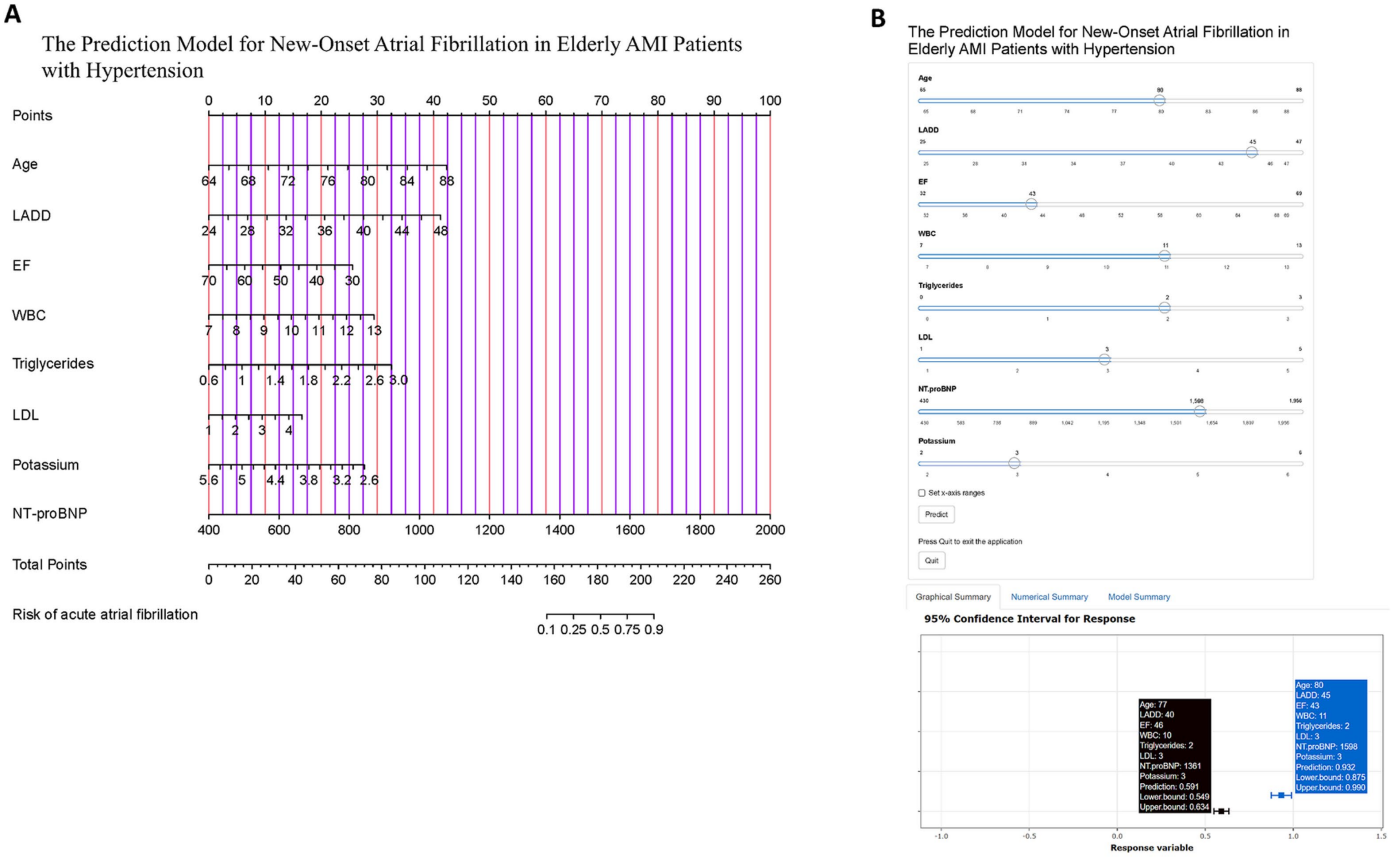
Construction and visualization of the predictive model for NOAF in elderly hypertensive AMI patients. **(A)** Nomogram based on eight variables (age, LADD, EF, WBC, triglycerides, LDL, NT-proBNP, and potassium) to estimate the probability of in-hospital NOAF. Each variable contributes to a total score, which corresponds to the predicted risk. **(B)** Dynamic online prediction interface developed using R Shiny, allowing real-time risk calculation based on individual patient input. Lower panel shows the 95% confidence interval for predicted risk. The link to access the tool is: https://longmao.shinyapps.io/NOAF/.

Based on the intersecting variables identified, we performed multivariable logistic regression analysis and constructed a nomogram model to predict the probability of NOAF in older adults with hypertension who experienced AMI (Figure 3). To further enhance the clinical usability and real-time interactivity of the model, we developed a web-based dynamic nomogram using the “DynNom” package in R and the Shiny platform. Users can input individual variable values and receive the predicted probability of NOAF in real-time. The link to access the tool is: https://longmao.shinyapps.io/NOAF/.

**Figure 4:**
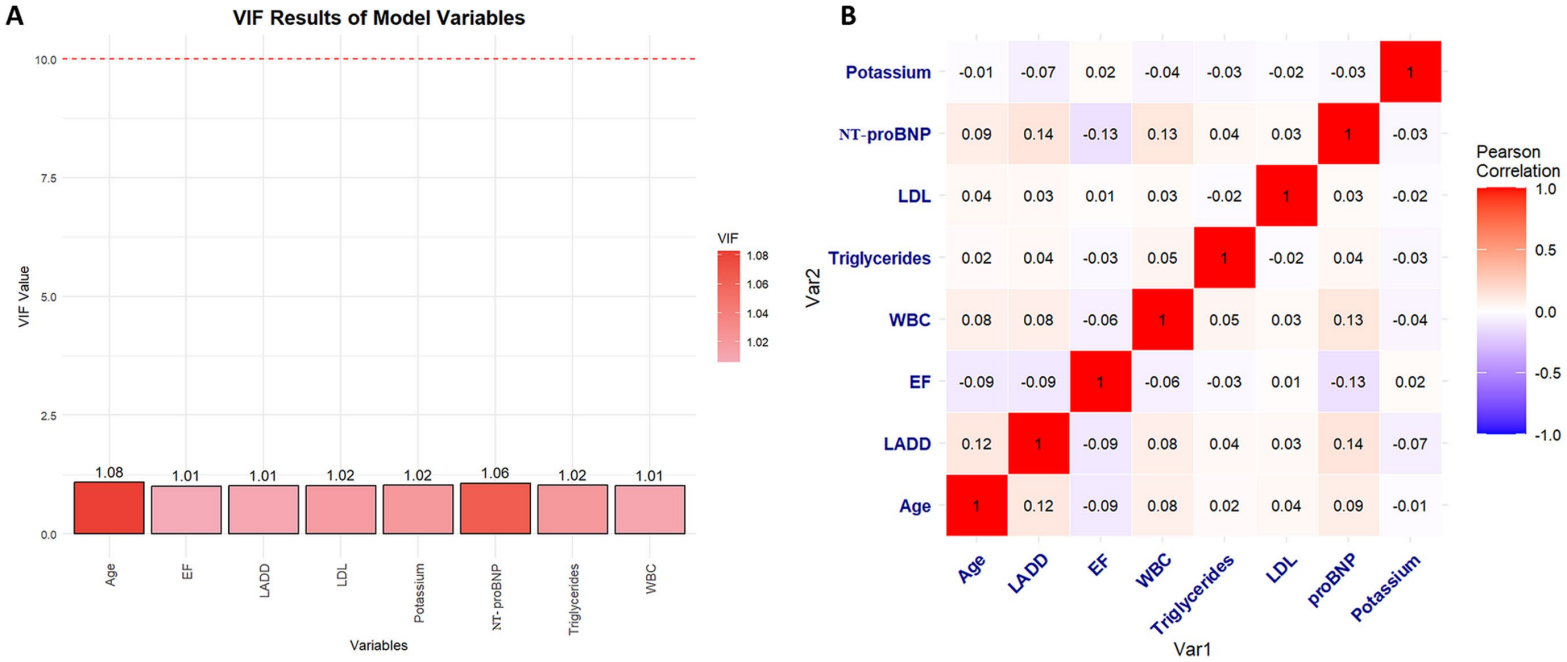
Multicollinearity assessment among selected predictors. **(A)** Variance inflation factor analysis showed that all variables had VIF values below 1.1, indicating no significant multicollinearity. **(B)** Pearson correlation matrix among the eight predictors demonstrated low pairwise correlations, further confirming the independence of variables used in the final model.

We calculated the VIF for each variable in the model, and the results indicated that all predictor variables had VIF values well below the threshold of 5. Specifically, the VIF values were: Age 1.08, LADD 1.01, EF 1.01, WBC 1.01, LDL 1.02, Triglycerides 1.02, NT-proBNP 1.06, Potassium 1.02 (Figure 4A). Additionally, we used a Pearson correlation matrix to further analyze the correlations between the variables (Figure 4B).

**Figure 5:**
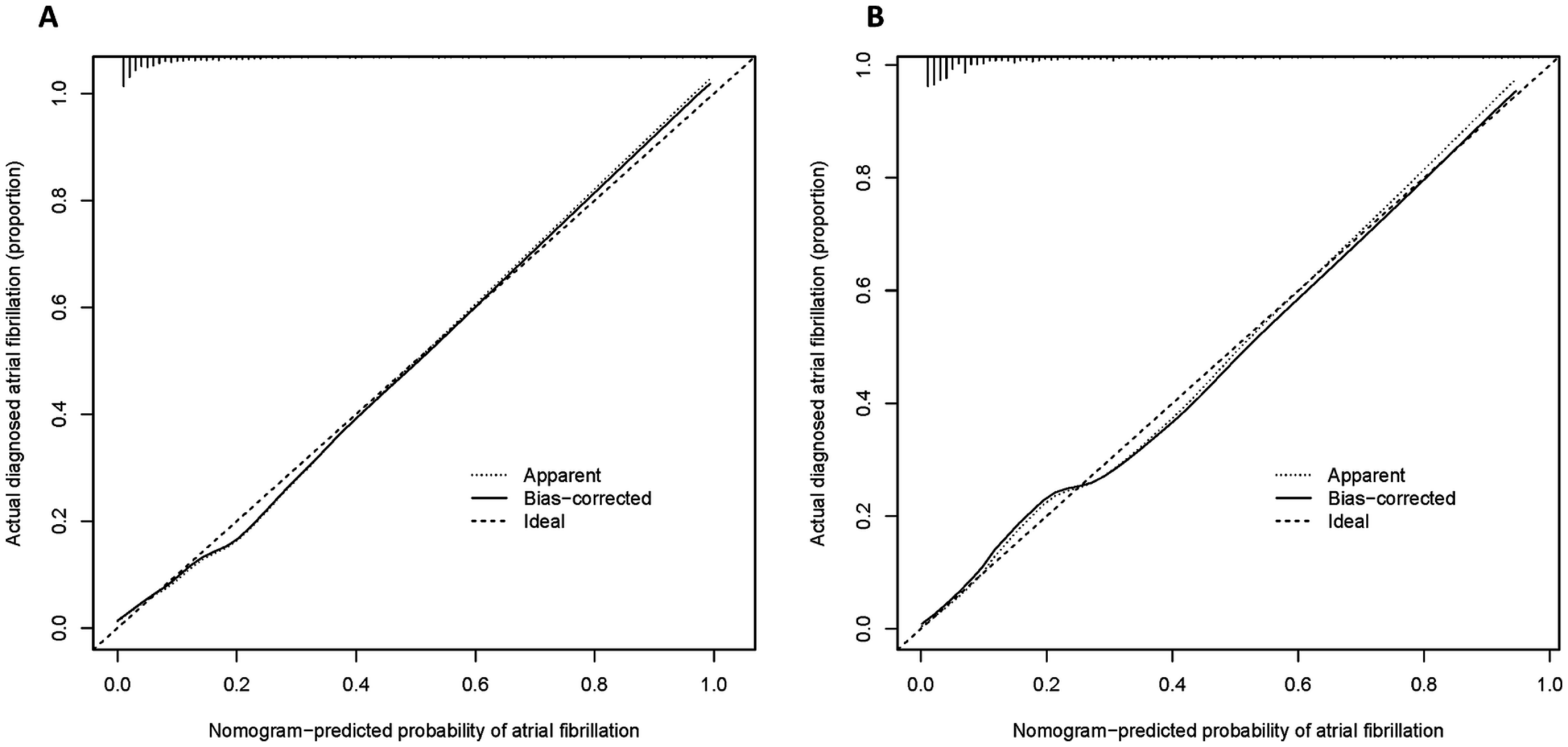
Calibration curves for the nomogram-predicted risk of NOAF. **(A)** Calibration plot in the training set; **(B)** calibration plot in the validation set. Both plots show good agreement between predicted and observed probabilities. The solid line represents bias-corrected estimates via bootstrap resampling (1,000 iterations), the dotted line shows apparent performance, and the dashed line indicates the ideal reference line.

**Figure 6:**
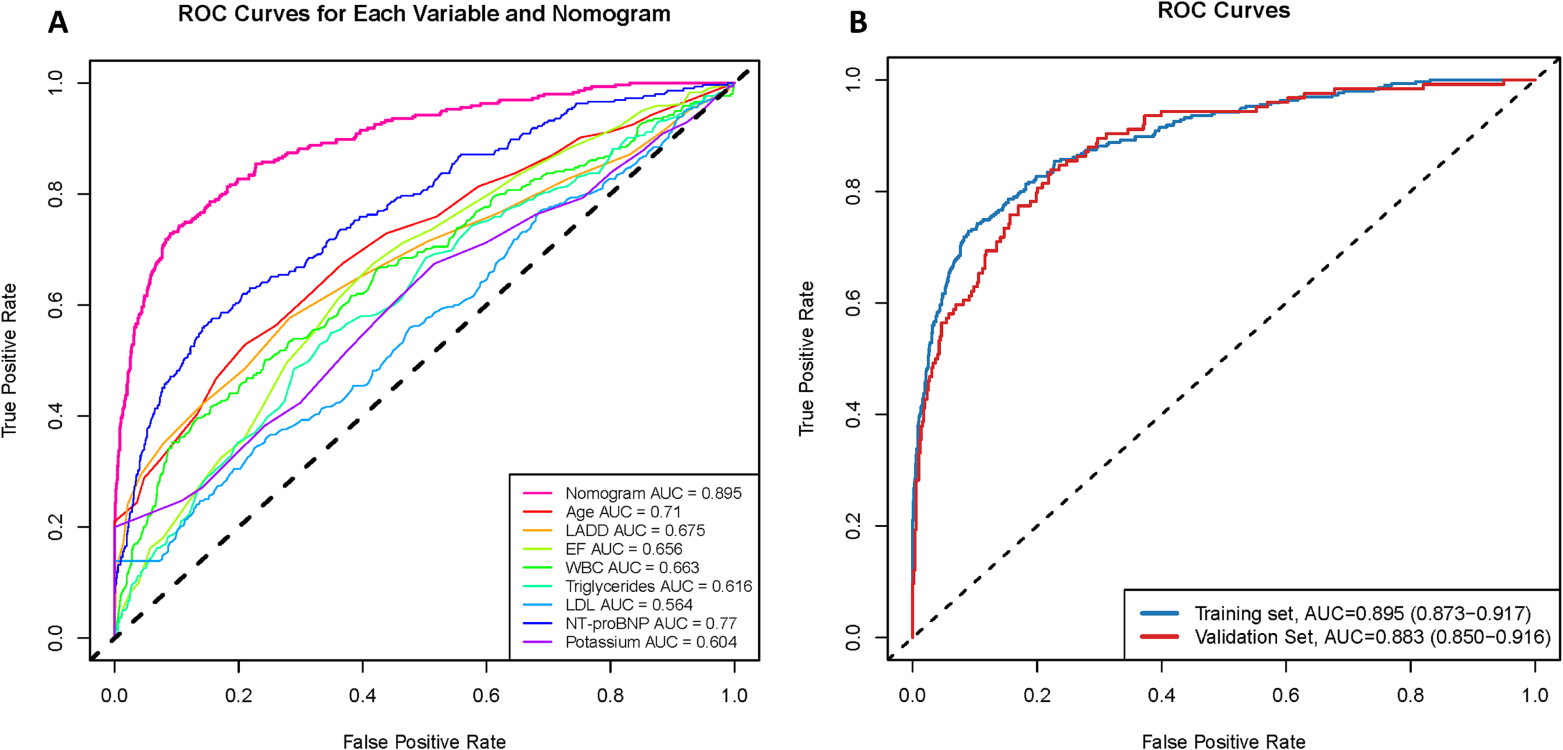
Discrimination performance of the NOAF prediction model. **(A)** ROC curves for each individual variable and the overall nomogram in the training set. The nomogram outperformed all single predictors, achieving an AUC of 0.895. **(B)** ROC curves of the nomogram in both training and validation sets, with AUCs of 0.895 and 0.883, respectively, indicating promising model discrimination and generalizability.

To comprehensively assess the predictive performance of the nomogram model, we employed the Bootstrap method with 1,000 repeated samples to evaluate the model’s calibration. The calibration curve of the model showed excellent fit between predicted and observed probabilities, with only slight deviations in the high-risk range (Figure 5), indicating that the model has good calibration ability and can accurately reflect the actual risk of NOAF at different probability levels. We then plotted the ROC curve and calculated the AUC in both the training and validation sets to evaluate the model’s discriminative ability. The results showed that in the training set, the nomogram model had an AUC of 0.895, the highest among multiple candidate variables (Figure 6A), while in the validation set, the AUC remained at 0.883 (95% confidence interval: 0.850–0.916) (Figure 6B), confirming the model’s generalizability and stability across different datasets. Furthermore, the C-statistic was also 0.895, further supporting the model’s effectiveness and consistency in identifying individuals at risk for NOAF.

**Figure 7:**
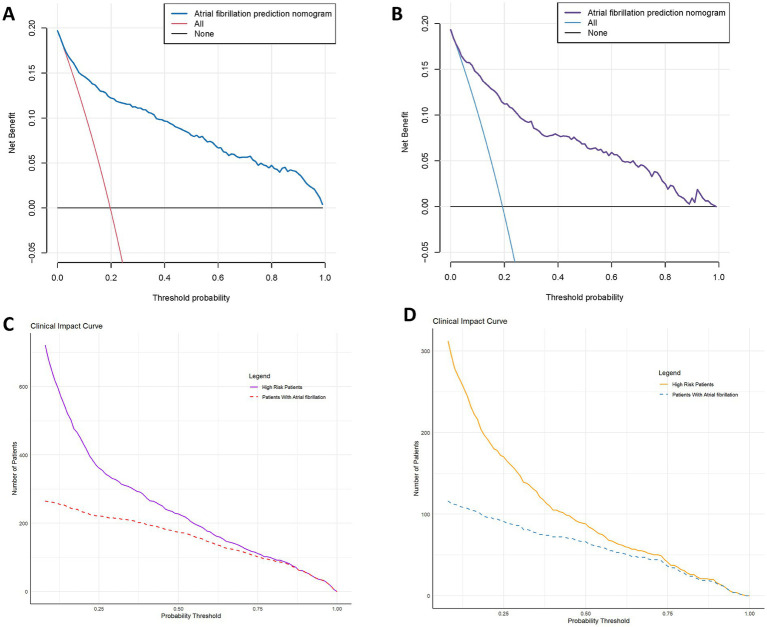
Clinical utility evaluation of the NOAF prediction model. **(A,B)** Decision curve analysis for the training set **(A)** and validation set **(B)**. The nomogram model provides higher net clinical benefit across a wide range of threshold probabilities compared to “treat all” or “treat none” strategies. **(C,D)** Clinical impact curves for the training set **(C)** and validation set **(D)**, illustrating the number of high-risk patients identified at different thresholds and the actual number of NOAF cases. The curves demonstrate the model’s practical effectiveness in population-level risk stratification.

To evaluate the clinical utility of the model, we used DCA to quantify the net benefit of the model at different risk thresholds. In both the training and validation sets, the model’s net benefit was significantly superior to the “treat all” or “treat none” assumptions. The clinical net benefit curves covered risk threshold ranges of 8–100% and 7–99%, respectively (Figures 7A,B), demonstrating the model’s potential decision-support value across a wide range of clinical scenarios. Additionally, we plotted CIC to assess the model’s effectiveness in population screening at different prediction thresholds (Figures 7C,D). The results showed that the model could accurately identify high-risk populations across various risk thresholds, with promising positive predictive ability and high clinical utility, providing quantitative support for early prevention of NOAF.

### Model explanation

**Figure 8:**
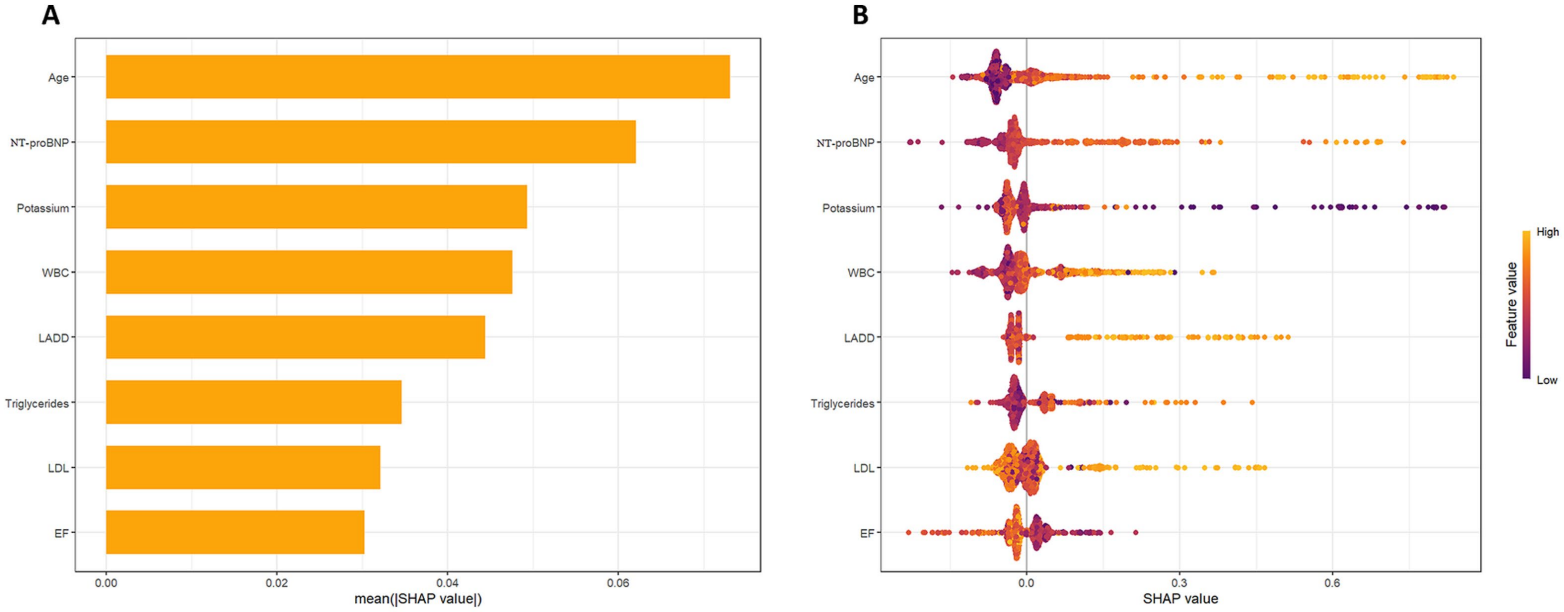
SHAP analysis of feature importance and contribution in the NOAF prediction model. **(A)** SHAP bar plot showing the average importance (mean SHAP value) of each predictor. Age, NT-proBNP, and potassium were the most influential variables. **(B)** SHAP bee swarm plot visualizing the impact of each feature across patients. Color gradient indicates the original feature values (from low to high), while SHAP value reflects each variable’s directional effect on model output.

To further enhance the model’s interpretability and clinical utility, we conducted both global and individual-level interpretive analysis of the eight key variables incorporated into the nomogram model using SHAP values. The SHAP algorithm quantifies the marginal contribution of each feature to the model’s output risk without compromising prediction performance. It also reveals the direction and intensity of each feature’s effect. As shown in Figure 8A, the SHAP mean bar plot illustrates the importance ranking of each variable in predicting the risk of NOAF in older adults with hypertension hospitalized for AMI. Among them, Age contributed the most to the model’s prediction, indicating that higher age was associated with an increased predicted probability of NOAF. NT-proBNP and Potassium followed closely, suggesting that markers of cardiac load and electrolyte status make major contributions to the model’s prediction of NOAF. WBC, LADD, triglycerides, LDL, and EF also exhibited significant influence, reflecting that these domains (inflammation, structural remodeling, lipid metabolism, and cardiac function) are informative for NOAF risk prediction in our cohort.

Additionally, we generated the SHAP bee swarm plot (Figure 8B) to visualize feature contributions. It should be noted that SHAP values quantify each feature’s contribution to the model’s predictions and do not imply causality. The color coding (from purple to yellow) represents the original values of each feature, while the horizontal axis quantifies their positive or negative impact on the predicted output. It can be observed that features such as Age, NT-proBNP, and Potassium significantly increase the predicted risk at higher levels, with their higher values concentrated on the right side of the SHAP values. This pattern is directionally consistent with prior clinical understanding of AF risk factors; however, SHAP values reflect contributions to prediction rather than causal or pathophysiological dominance. In contrast, EF shows a positive SHAP distribution at lower values, indicating that impaired cardiac function is a significant predictor of NOAF. Triglycerides and LDL, on the other hand, display moderate contributions with some heterogeneity in their values.

## Discussion

This study developed and internally validated a multifactorial model to predict the risk of NOAF in older adults with hypertension hospitalized for AMI, incorporating the SHAP algorithm to enhance model interpretability. In our cohort, the in-hospital incidence of NOAF was 19.6%, which is consistent with previous reports in general AMI populations (11.6–18.9%) but slightly higher than that observed in non-hypertensive populations (6, 17), suggesting that hypertension may further increase NOAF risk. Although prior studies have examined individual predictors of NOAF in AMI patients—such as age, left atrial structure, and NT-proBNP—systematic predictive modeling focused on older hypertensive patients with AMI remains limited, particularly models integrating inflammation, metabolism, electrolyte balance, and cardiac function. To address this gap, we provide an in-depth supplement and expansion: including Boruta, LASSO, together with univariate logistic regression screening and intersection analysis, we identified and incorporated eight key variables (age, LADD, EF, WBC, triglycerides, LDL, NT-proBNP, potassium) into a multivariable logistic regression–based nomogram model. These variables capture complementary domains relevant to NOAF risk, including structural remodeling, cardiac function, metabolic status, inflammation, and electrolyte homeostasis. A web-based dynamic nomogram was further developed to facilitate individualized risk estimation in clinical settings.^1^ The model demonstrated promising and consistent internal discrimination, with AUCs of 0.895 in the training set and 0.883 in the validation set. Several clinical risk scores for AF or NOAF, including the C2HEST score, have been reported to show heterogeneous performance across populations (11). In our validation cohort, a modified C2HEST score (excluding the thyroid disease component due to unavailable data) showed limited discrimination (AUC = 0.667), whereas the proposed model achieved higher discrimination (AUC = 0.883), supporting the incremental value of a population-specific, machine learning–assisted prediction approach in older hypertensive patients with AMI. Model calibration and potential clinical utility were further assessed using bootstrap resampling, calibration curves, and DCA. Importantly, the threshold probabilities applied in DCA should be interpreted as reflecting different clinical risk–benefit preferences rather than fixed intervention cut-offs. In the absence of guideline-recommended thresholds for NOAF prevention, lower thresholds may correspond to enhanced rhythm surveillance, whereas higher thresholds may be relevant for more intensive preventive strategies. Across a wide and clinically plausible range of thresholds, the model consistently provided greater net benefit than “treat-all” or “treat-none” strategies, suggesting its potential role as a flexible decision-support tool rather than a prescriptive rule based on a single probability cut-off. Finally, the integration of SHAP analysis allowed transparent quantification of each variable’s contribution to model predictions at both global and individual levels. Consistent with best practice, SHAP-based importance reflects predictive contribution rather than causal or mechanistic dominance. Overall, by combining machine learning–assisted feature screening with a multivariable logistic regression–based nomogram and interpretable SHAP analysis, this study provides a structured and internally validated approach for NOAF risk stratification in older adults with hypertension hospitalized for AMI.

Age is a well-established risk factor for atrial fibrillation and demonstrated particularly strong predictive value in the present population. Aging is associated with progressive degenerative changes in cardiac structure and function, including atrial fibrosis, reduced tissue elasticity, impaired electrical conduction, and altered autonomic regulation, all of which contribute to decreased electrical stability of atrial myocytes and increased susceptibility to AF (18). In older individuals with hypertension, these age-related alterations are further amplified. Long-standing hypertension promotes vascular stiffness, left ventricular hypertrophy, and sustained elevation of left atrial pressure, accelerating atrial structural remodeling and fibrosis (19, 20). In addition, hypertension-induced shear stress and atrial endocardial microinjury enhance local inflammatory activation and collagen deposition, further exacerbating atrial electrical–structural remodeling (21). Under the combined effects of aging, hypertension, and the acute ischemic and inflammatory stress of AMI, atrial substrate vulnerability is markedly increased, predisposing this population to NOAF during hospitalization. Consistent with previous reports identifying age as an independent risk factor for NOAF after AM (22, 23). Our findings underscore its particularly prominent predictive role in older hypertensive patients. Thus, age in this context reflects not only chronological aging but also cumulative atrial remodeling burden, highlighting the importance of vigilant NOAF risk assessment in this high-risk group.

LADD, as a marker of left atrial structural remodeling, emerged as a key predictor of NOAF in older adults with hypertension hospitalized for AMI. Chronic hypertension leads to sustained elevation of left ventricular filling pressure, which is transmitted to the left atrium, resulting in prolonged pressure overload and progressive atrial dilation (24). Over time, reduced atrial compliance, wall thickening, and fibrosis contribute to an increase in LADD. Structural remodeling of the left atrium provides a critical substrate for atrial fibrillation. Atrial fibrosis and collagen deposition prolong conduction pathways and increase electrical heterogeneity, facilitating reentrant activity (25). Concurrently, atrial enlargement is associated with hemodynamic alterations, inflammatory activation, and autonomic imbalance, further destabilizing atrial electrophysiology (26). Previous studies have consistently shown that increases in left atrial size are associated with higher AF risk, an effect that is further amplified in the presence of hypertension and myocardial infarction (27). In older hypertensive patients, limited atrial repair capacity and long-standing pressure overload render atrial remodeling less reversible. Superimposed acute ischemic stress during AMI may further precipitate electrical instability. Accordingly, LADD reflects cumulative atrial remodeling burden and serves as a clinically accessible indicator for identifying patients at increased risk of NOAF, supporting its value in early risk stratification and preventive management.

EF reflects left ventricular systolic function and overall pumping capacity and emerged as an independent predictor of NOAF in older adults with hypertension hospitalized for AMI. Reduced EF typically indicates more extensive myocardial injury and diminished functional reserve, which are particularly common in elderly patients following AMI. Chronic hypertension contributes to left ventricular hypertrophy, increased myocardial stiffness, and interstitial fibrosis, resulting in long-term impairment of both systolic and diastolic function. Superimposed acute myocardial necrosis during AMI further compromises ventricular contractility, leading to a decline in EF. Hemodynamically, reduced EF decreases stroke volume and elevates left atrial and pulmonary venous pressures, thereby promoting atrial dilation, structural remodeling, and electrophysiological instability (28). In addition, patients with impaired EF frequently exhibit heart failure and adverse cardiac remodeling, accompanied by activation of neurohormonal pathways such as the renin–angiotensin–aldosterone system and sympathetic nervous system, which further aggravate myocardial fibrosis and atrial electrical disturbances (29). Notably, ventricular dysfunction may contribute differently to NOAF phenotypes. Early NOAF following AMI is often related to acute ischemia, inflammation, and transient hemodynamic instability, whereas late NOAF is more likely to reflect sustained ventricular–atrial interaction and progressive atrial remodeling in the setting of chronic systolic dysfunction (30). Accordingly, EF serves as an integrative marker linking ventricular injury, atrial load, and arrhythmic vulnerability. From a clinical perspective, dynamic assessment and optimization of ventricular function may aid both heart failure management and NOAF risk stratification in older hypertensive patients with AMI.

WBC reflects systemic inflammatory and immune activation and emerged as an informative predictor of NOAF in older adults with hypertension hospitalized for AMI. Acute myocardial infarction is commonly accompanied by a systemic inflammatory response, resulting in elevated white blood cell levels (31). Inflammatory activation contributes not only to plaque instability but also to atrial electrophysiological alterations through cytokine release and oxidative stress, thereby increasing susceptibility to atrial fibrillation (32). In hypertensive individuals, chronic low-grade inflammation and endothelial dysfunction may further amplify this inflammatory response, particularly in older patients with limited inflammatory reserve, predisposing them to arrhythmic events. Elevated WBC has also been associated with adverse in-hospital and long-term outcomes in AMI, underscoring its role as a marker of disease severity and inflammatory burden (33, 34). Importantly, inflammatory activity may differentially contribute to NOAF phenotypes. Early NOAF is more frequently associated with acute ischemic injury and inflammation, whereas late NOAF is more likely to reflect persistent atrial remodeling and fibrosis, leading to distinct arrhythmic risk profiles (35). These findings suggest that WBC primarily captures inflammation-related vulnerability, which may be particularly relevant for early-phase NOAF risk assessment and clinical monitoring.

Triglycerides and LDL are key indicators of disordered lipid metabolism and were identified as relevant predictors of NOAF in older hypertensive patients with AMI. Dyslipidemia accelerates atherosclerosis, promotes coronary plaque instability, and contributes to myocardial ischemia and its complications. Elevated lipid levels have been associated with endothelial dysfunction, inflammation, and oxidative stress, processes that may facilitate adverse cardiac remodeling and increase arrhythmic susceptibility (36). In patients with longstanding hypertension, chronic vascular stress further promotes lipid deposition and impairs coronary microcirculation. In older adults, age-related metabolic alterations and arterial stiffness often exacerbate lipid abnormalities, while elevated triglycerides may contribute to microvascular dysfunction and metabolic stress, increasing atrial vulnerability. Abnormal triglyceride and LDL levels have also been linked to a higher incidence of atrial fibrillation, potentially reflecting shared inflammatory and oxidative stress pathways involved in AF pathogenesis (37). Collectively, these findings suggest that lipid-related metabolic and inflammatory disturbances represent an important substrate for NOAF risk, underscoring the potential value of lipid profile assessment in risk stratification of older hypertensive patients hospitalized with AMI.

NT-proBNP is a neurohormonal biomarker released in response to myocardial pressure and volume overload and is widely used to assess cardiac dysfunction. In older hypertensive patients hospitalized for AMI, elevated NT-proBNP levels reflect the combined effects of chronic pressure overload, ventricular hypertrophy, atrial enlargement, and acute ischemic injury. Long-standing hypertension increases cardiac afterload and ventricular stiffness, while AMI-related myocardial necrosis further impairs ventricular function, leading to marked intracardiac pressure elevation and NT-proBNP release. Beyond its role as a marker of heart failure, NT-proBNP has been consistently associated with atrial fibrillation, particularly in the context of atrial structural remodeling, excessive volume load, and increased atrial pressure (38). In older individuals with hypertension, the coexistence of chronic hemodynamic stress and acute myocardial injury may therefore amplify NT-proBNP elevation, reflecting limited cardiac compensation and reduced atrial electrical stability. Accordingly, NT-proBNP serves as an informative indicator for NOAF risk stratification and for monitoring cardiac volume status in high-risk patients.

Potassium is a key electrolyte for maintaining myocardial electrical stability, and disturbances in potassium homeostasis are closely linked to arrhythmogenesis. In this study, lower potassium levels were identified as an important risk factor for NOAF in older adults with hypertension and AMI. Hypertensive patients are particularly susceptible to hypokalemia due to diuretic use, renal dysfunction, and impaired homeostatic regulation. Hypokalemia reduces myocardial resting membrane potential, prolongs repolarization, and increases atrial automaticity, thereby facilitating ectopic activity and reentrant circuits that predispose to atrial fibrillation (39, 40). In the setting of AMI, age-related renal impairment, neurohormonal activation, and acute metabolic disturbances may further exacerbate potassium fluctuations. Consistent with prior evidence, maintaining potassium levels within the physiological range is crucial for reducing NOAF risk and stabilizing cardiac electrophysiology in this high-risk population (41). Therefore, in clinical settings, closely monitoring and promptly correcting potassium levels in high-risk populations is of great significance for reducing the occurrence of atrial fibrillation, maintaining stable cardiac electrical activity, and optimizing overall prognosis.

## Limitations

Although this study has made valuable progress in the development and interpretability enhancement of the NOAF prediction model for a specifically defined high-risk population, namely older adults with hypertension hospitalized for AMI, several limitations remain. First, this study was intentionally designed as a single-center retrospective cohort focusing exclusively on elderly hypertensive patients with AMI who underwent coronary angiography, with all sample data sourced from Qinhuangdao First Hospital. While this strict inclusion strategy enhances internal validity within this high-risk subgroup, it inherently limits the external applicability and generalizability of the model. Importantly, the current model is not intended for use in younger patients, non-hypertensive individuals, or AMI patients managed conservatively, and its application outside this narrowly defined population is not supported by the present data. Future studies should incorporate multi-center, large-sample prospective cohorts to validate the model’s stability and universality within similar high-risk populations, and to explore whether recalibration or re-development is required for other clinical settings. Second, although the study included a wide range of clinical and laboratory variables, some factors that may significantly impact NOAF risk, such as specific drug treatments (e.g., antihypertensive drugs, antiarrhythmic drugs), detailed electrocardiogram parameters, and patient lifestyle and socioeconomic status, were not systematically included or analyzed due to data limitations. This could limit the model’s predictive capability. Third, as a retrospective study, information bias is inevitable during data collection, with some key variables having missing or measurement errors. In addition, outcome ascertainment relied on in-hospital electrocardiographic monitoring performed as part of routine clinical care, and the intensity and duration of rhythm monitoring were not standardized. Consequently, detection bias cannot be excluded, as patients with more severe clinical conditions may have undergone more intensive monitoring, increasing the likelihood of NOAF detection. This limitation may partly explain the observed associations between NOAF and biomarkers reflecting disease severity, such as NT-proBNP or inflammatory indices. Future prospective studies with standardized rhythm monitoring protocols are warranted to mitigate this bias. Fourth, while this study utilized machine learning–assisted methods for feature selection, it primarily relied on logistic regression. The potential of other advanced machine learning or deep learning algorithms (such as XGBoost, CatBoost, neural networks) in NOAF risk prediction was not systematically compared, limiting the exploration of the model’s optimal performance. Fifth, although internal validation was performed using a random 70:30 data split and bootstrap resampling, this study did not include true external validation based on independent cohorts. Therefore, the reported performance metrics may still be subject to optimism, and the generalizability of the model across different geographic regions, healthcare systems, and patient populations cannot be fully established. Independent external validation in geographically and clinically distinct cohorts is required before routine clinical implementation can be considered. Sixth, although SHAP methods were introduced to enhance model interpretability, the model’s user-friendliness in real-world clinical settings, its feasibility for integration into hospital information systems, and its actual impact on clinical decision-making behavior have not yet been systematically evaluated. Moreover, this study did not distinguish between early and late NOAF. Early NOAF, typically occurring shortly after acute myocardial infarction, may be driven by acute ischemia, inflammation, and transient hemodynamic instability, whereas late NOAF is more likely to reflect advanced atrial remodeling and fibrosis, with potentially different prognostic implications. The lack of temporal stratification may therefore limit the interpretation of risk patterns captured by the model. Future multicenter prospective studies are warranted to externally validate the model, explore temporal NOAF phenotypes, incorporate more comprehensive clinical and biological variables, and facilitate integration into routine clinical pathways, thereby improving both scientific rigor and clinical utility.

## Conclusion

This study, based on a large sample single-center retrospective cohort, systematically developed and validated a predictive model for the risk of NOAF during hospitalization in older adults with hypertension and AMI. Through multi-algorithm feature selection, we integrated key clinical variables to build a robust nomogram model, and successfully developed a dynamic web-based tool. The model demonstrated high discriminative ability and strong calibration in both the training and validation sets. Notably, this study is the first to incorporate the SHAP interpretability algorithm into the construction and interpretation of the NOAF risk prediction model for older adults with hypertension and AMI. It provides quantitative explanations of the model’s predictions at both global and individual levels, significantly enhancing the model’s transparency and clinical trustworthiness. The comprehensive analysis of multidimensional variables revealed the key mechanisms underlying the occurrence of NOAF in this high-risk population, offering theoretical support for personalized early warning and precise interventions.

## Data Availability

The raw data supporting the conclusions of this article will be made available by the authors, without undue reservation.

## Footnotes

1
https://longmao.shinyapps.io/NOAF/

## References

[1] GBD 2021 Diseases and Injuries Collaborators. Global incidence, prevalence, years lived with disability (YLDs), disability-adjusted life-years (DALYs), and healthy life expectancy (HALE) for 371 diseases and injuries in 204 countries and territories and 811 subnational locations, 1990-2021: a systematic analysis for the global burden of disease study 2021. Lancet. (2024) 403:2133–61. doi: 10.1016/S0140-6736(24)00757-8, 38642570 PMC11122111

[2] Boersma E Mercado N Poldermans D Gardien M Vos J Simoons ML. Acute myocardial infarction. Lancet. (2003) 361:847–58. doi: 10.1016/S0140-6736(03)12712-2, 12642064

[3] Wang MC Lloyd-Jones DM. Cardiovascular risk assessment in hypertensive patients. Am J Hypertens. (2021) 34:569–77. doi: 10.1093/ajh/hpab021 33503227

[4] Fan J Watanabe T. Atherosclerosis: known and unknown. Pathol Int. (2022) 72:151–60. doi: 10.1111/pin.13202 35076127

[5] Erne P Radovanovic D Schoenenberger AW Bertel O Kaeslin T Essig M . Impact of hypertension on the outcome of patients admitted with acute coronary syndrome. J Hypertens. (2015) 33:860–7. doi: 10.1097/HJH.0000000000000343 25915891

[6] Luo J Xu S Li H Gong M Li Z Liu B . Long-term impact of the burden of new-onset atrial fibrillation in patients with acute myocardial infarction: results from the NOAFCAMI-SH registry. Europace. (2021) 23:196–204. doi: 10.1093/europace/euaa234, 32929491

[7] Garside T Bedford JP Vollam S Gerry S Rajappan K Watkinson PJ. Increased long-term mortality following new-onset atrial fibrillation in the intensive care unit: a systematic review and meta-analysis. J Crit Care. (2022) 72:154161. doi: 10.1016/j.jcrc.2022.154161, 36215944

[8] Joglar JA Chung MK Armbruster AL Benjamin EJ Chyou JY Cronin EM . 2023 ACC/AHA/ACCP/HRS guideline for the diagnosis and management of atrial fibrillation: a report of the american college of cardiology/american heart association joint committee on clinical practice guidelines. Circulation. (2024) 149:e1–e156. doi: 10.1161/CIR.0000000000001193, 38033089 PMC11095842

[9] Pirruccello JP Di Achille P Choi SH Rämö JT Khurshid S Nekoui M . Deep learning of left atrial structure and function provides link to atrial fibrillation risk. Nat Commun. (2024) 15:4304. doi: 10.1038/s41467-024-48229-w, 38773065 PMC11109224

[10] Avci BK Gulmez O Donmez G Pehlivanoglu S. Early changes in atrial electromechanical coupling in patients with hypertension: assessment by tissue doppler imaging. Chin Med J. (2016) 129:1311–5. doi: 10.4103/0366-6999.182846, 27231168 PMC4894041

[11] Pastori D Menichelli D Li YG Brogi T Biccirè FG Pignatelli P . Usefulness of the c(2)HEST score to predict new onset atrial fibrillation. A systematic review and meta-analysis on >11 million subjects. Eur J Clin Investig. (2024) 54:e14293. doi: 10.1111/eci.14293, 39072756

[12] Rudin C. Stop explaining black box machine learning models for high stakes decisions and use interpretable models instead. Nat Mach Intell. (2019) 1:206–15. doi: 10.1038/s42256-019-0048-x, 35603010 PMC9122117

[13] Karim MR Islam T Shajalal M Beyan O Lange C Cochez M . Explainable AI for bioinformatics: methods, tools and applications. Brief Bioinform. (2023) 24:bbad236. doi: 10.1093/bib/bbad236 37478371

[14] Gulati M Levy PD Mukherjee D Amsterdam E Bhatt DL Birtcher KK . 2021 AHA/ACC/ASE/CHEST/SAEM/SCCT/SCMR guideline for the evaluation and diagnosis of chest pain: a report of the american college of cardiology/american heart association joint committee on clinical practice guidelines. Circulation. (2021) 144:e368–454. doi: 10.1161/CIR.0000000000001029, 34709879

[15] Byrne RA Rossello X Coughlan JJ Barbato E Berry C Chieffo A . 2023 ESC guidelines for the management of acute coronary syndromes. Eur Heart J. (2023) 44:3720–826. doi: 10.1093/eurheartj/ehad191, 37622654

[16] McIntyre WF Um KJ Cheung CC Belley-Côté EP Dingwall O Devereaux PJ . Atrial fibrillation detected initially during acute medical illness: a systematic review. Eur Heart J Acute Cardiovasc Care. (2019) 8:130–41. doi: 10.1177/2048872618799748 30403364

[17] Hao C Luo J Liu B Xu W Li Z Gong M . Prognostic significance of new-onset atrial fibrillation in heart failure with preserved, mid-range, and reduced ejection fraction following acute myocardial infarction: data from the NOAFCAMI-SH registry. Clin Interv Aging. (2022) 17:479–93. doi: 10.2147/CIA.S358349, 35444413 PMC9014224

[18] Donato AJ Machin DR Lesniewski LA. Mechanisms of dysfunction in the aging vasculature and role in age-related disease. Circ Res. (2018) 123:825–48. doi: 10.1161/CIRCRESAHA.118.312563, 30355078 PMC6207260

[19] Tsioufis C Konstantinidis D Nikolakopoulos I Vemmou E Kalos T Georgiopoulos G . Biomarkers of atrial fibrillation in hypertension. Curr Med Chem. (2019) 26:888–97. doi: 10.2174/0929867324666171006155516, 28990508

[20] Huang Y Zhao J Zhou Z Guo X Xu Y Huang T . Persistent hypertension induces atrial remodeling and atrial fibrillation through DNA damage and ATM/CHK2/p53 signaling pathway. Biochim Biophys Acta (BBA) - Mol Basis Dis. (2025) 1871:167534. doi: 10.1016/j.bbadis.2024.167534, 39366645

[21] Durante A Mazzapicchi A Baiardo Redaelli M. Systemic and cardiac microvascular dysfunction in hypertension. Int J Mol Sci. (2024) 25:13294. doi: 10.3390/ijms252413294, 39769057 PMC11677602

[22] Li Z Liu Q Liu F Hidru TH Yang Y Wang S . Atrial cardiomyopathy markers and new-onset atrial fibrillation risk in patients with acute myocardial infarction. Eur J Intern Med. (2022) 102:72–9. doi: 10.1016/j.ejim.2022.04.019, 35513991

[23] Dal Zotto B Barbieri L Tumminello G Saviano M Gentile D Lucreziotti S . New onset atrial fibrillation in STEMI patients: main prognostic factors and clinical outcome. Diagnostics. (2023) 13:613. doi: 10.3390/diagnostics13040613, 36832101 PMC9955053

[24] Kockskamper J Pluteanu F. Left atrial myocardium in arterial hypertension. Cells. (2022) 11:11. doi: 10.3390/cells11193157, 36231118 PMC9563039

[25] Nattel S. Molecular and cellular mechanisms of atrial fibrosis in atrial fibrillation. JACC Clin Electrophysiol. (2017) 3:425–35. doi: 10.1016/j.jacep.2017.03.002, 29759598

[26] Qiu D Peng L Ghista DN Wong KKL. Left atrial remodeling mechanisms associated with atrial fibrillation. Cardiovasc Eng Technol. (2021) 12:361–72. doi: 10.1007/s13239-021-00527-w, 33650086

[27] Parkash R Green MS Kerr CR Connolly SJ Klein GJ Sheldon R . The association of left atrial size and occurrence of atrial fibrillation: a prospective cohort study from the canadian registry of atrial fibrillation. Am Heart J. (2004) 148:649–54. doi: 10.1016/j.ahj.2004.04.029, 15459596

[28] Wolsk E Kaye DM Komtebedde J Shah SJ Borlaug BA Burkhoff D . Determinants and consequences of heart rate and stroke volume response to exercise in patients with heart failure and preserved ejection fraction. Eur J Heart Fail. (2021) 23:754–64. doi: 10.1002/ejhf.2146, 33686716

[29] Hartupee J Mann DL. Neurohormonal activation in heart failure with reduced ejection fraction. Nat Rev Cardiol. (2017) 14:30–8. doi: 10.1038/nrcardio.2016.163, 27708278 PMC5286912

[30] Biccirè FG Pastori D Torromeo C Acconcia MC Capone S Ferrari I . Acute atrial ischemia associates with early but not late new-onset atrial fibrillation in STEMI patients treated with primary PCI: relationship with in-hospital outcomes. J Cardiol. (2021) 78:368–74. doi: 10.1016/j.jjcc.2021.05.013, 34130874

[31] Fang L Moore XL Dart AM Wang LM. Systemic inflammatory response following acute myocardial infarction. J Geriatr Cardiol. (2015) 12:305–12. doi: 10.11909/j.issn.1671-5411.2015.03.020, 26089856 PMC4460175

[32] Yao Y Yang M Liu D Zhao Q. Immune remodeling and atrial fibrillation. Front Physiol. (2022) 13:927221. doi: 10.3389/fphys.2022.927221, 35936905 PMC9355726

[33] Grzybowski M Welch RD Parsons L Ndumele CE Chen E Zalenski R . The association between white blood cell count and acute myocardial infarction in-hospital mortality: findings from the national registry of myocardial infarction. Acad Emerg Med. (2004) 11:1049–60. doi: 10.1197/j.aem.2004.06.005, 15466147

[34] Dragu R Huri S Zukermann R Suleiman M Mutlak D Agmon Y . Predictive value of white blood cell subtypes for long-term outcome following myocardial infarction. Atherosclerosis. (2008) 196:405–12. doi: 10.1016/j.atherosclerosis.2006.11.022, 17173924

[35] Shiyovich A Axelrod M Gilutz H Plakht Y. Early versus late new-onset atrial fibrillation in acute myocardial infarction: differences in clinical characteristics and predictors. Angiology. (2019) 70:921–8. doi: 10.1177/0003319719867542, 31387358

[36] Gianazza E Brioschi M Martinez Fernandez A Casalnuovo F Altomare A Aldini G . Lipid peroxidation in atherosclerotic cardiovascular diseases. Antioxid Redox Signal. (2021) 34:49–98. doi: 10.1089/ars.2019.7955, 32640910

[37] Lee HJ Lee SR Choi EK Han KD Oh S. Low lipid levels and high variability are associated with the risk of new-onset atrial fibrillation. J Am Heart Assoc. (2019) 8:e012771. doi: 10.1161/JAHA.119.012771, 31771440 PMC6912974

[38] Buttner P Schumacher K Dinov B Zeynalova S Sommer P Bollmann A . Role of NT-proANP and NT-proBNP in patients with atrial fibrillation: association with atrial fibrillation progression phenotypes. Heart Rhythm. (2018) 15:1132–7. doi: 10.1016/j.hrthm.2018.03.021, 29604419

[39] Tazmini K Frisk M Lewalle A Laasmaa M Morotti S Lipsett DB . Hypokalemia promotes arrhythmia by distinct mechanisms in atrial and ventricular myocytes. Circ Res. (2020) 126:889–906. doi: 10.1161/CIRCRESAHA.119.315641, 32070187 PMC7098435

[40] Weiss JN Qu Z Shivkumar K. Electrophysiology of hypokalemia and hyperkalemia. Circ Arrhythm Electrophysiol. (2017) 10:10. doi: 10.1161/CIRCEP.116.004667, 28314851 PMC5399982

[41] O'Brien B Campbell NG Allen E Jamal Z Sturgess J Sanders J . Potassium supplementation and prevention of atrial fibrillation after cardiac surgery: the TIGHT k randomized clinical trial. JAMA. (2024) 332:979–88. doi: 10.1001/jama.2024.17888, 39215972 PMC11366075

