# Supplementary material for: Development of a predictive model for in-hospital new-onset atrial fibrillation in older adults with hypertension and acute myocardial infarction, enhanced by SHAP interpretability: a retrospective cohort study
Source: Front Med (Lausanne). 2026 Mar 6;13:1747281. doi: 10.3389/fmed.2026.1747281 (PMC13002802; doi:10.3389/fmed.2026.1747281)
Supplement: Supplementary file 1 [file Table_1.docx]

| **Table S1 Baseline characteristics of patients with and without NOAF in the training cohort** | | | | |
| --- | --- | --- | --- | --- |
| **Variables** | **Total(N=1498)** | **NOAF (N=1203)** | **Non-NOAF (N=295)** | **p-value** |
| **Gender, N (%)** |  |  |  | **0.140** |
| **Male** | **816(54.5%)** | **644(53.5%)** | **172(58.3%)** |  |
| **Female** | **682(45.5%)** | **559(45.5%)** | **123(41.7%)** |  |
| **Age, mean±SD (years)** | **73.82±5.99** | **72.85±5.37** | **77.76±6.74** | **<0.001** |
| **BMI, mean±SD** | **24.54±2.29** | **24.53±2.29** | **24.58±2.31** | **0.725** |
| **Heart rate at admission, mean±SD** | **84.38±15.27** | **84.94±15.05** | **86.17±16.06** | **0.049** |
| **Systolic blood pressure at admission, mean±SD** | **147.48±21.86** | **146.87±22.16** | **149.96±20.41** | **0.021** |
| **Diastolic blood pressure at admission, mean±SD** | **79.68±12.49** | **79.32±12.44** | **81.15±12.58** | **0.040** |
| **Smoking history, N (%)** |  |  |  | **0.788** |
| **No** | **970(64.8%)** | **777(64.6%)** | **193(65.4%)** |  |
| **Yes** | **528(35.2%)** | **426(35.4%)** | **102(34.6%)** |  |
| **Drinking history, N (%)** |  |  |  | **0.606** |
| **No** | **1239(82.7%)** | **1239(82.7%)** | **247(83.7%)** |  |
| **Yes** | **259(17.3%)** | **259(17.3%)** | **48(16.3%)** |  |
| **Comorbidity, N (%)** |  |  |  |  |
| **Coronary heart disease** |  |  |  | **0.042** |
| **No** | **1072(71.6%)** | **875(72.7%)** | **197(66.8%)** |  |
| **Yes** | **426(28.4%)** | **328(27.3%)** | **98(33.2%)** |  |
| **Chronic renal failure** |  |  |  | **0.905** |
| **No** | **1461(97.5%)** | **1173(97.5%)** | **288(97.6%)** |  |
| **Yes** | **37(2.5%)** | **30(2.5%)** | **7(2.4%)** |  |
| **Diabetes** |  |  |  | **<0.001** |
| **No** | **1235(82.4%)** | **1014(84.3%)** | **221(74.9%)** |  |
| **Yes** | **263(17.6%)** | **189(15.7%)** | **74(25.1%)** |  |
| **Heart valve disease** |  |  |  | **0.029** |
| **No** | **1342(89.6%)** | **1088(90.4%)** | **254(86.1%)** |  |
| **Yes** | **156(10.4%)** | **115(9.6%)** | **41(13.9%)** |  |
| **Old cerebral infarction** |  |  |  | **0.854** |
| **No** | **1189(79.4%)** | **956(79.5%)** | **233(79.0%)** |  |
| **Yes** | **309(20.6%)** | **247(20.5%)** | **62(21.0%)** |  |
| **Cancer** |  |  |  | **0.479** |
| **No** | **1453(94.0%)** | **1165(96.8%)** | **288(97.6%)** |  |
| **Yes** | **45(3.0%)** | **38(3.2%)** | **7(2.4%)** |  |
| **COPD** |  |  |  | **0.276** |
| **No** | **1308(87.3%)** | **1056(87.8%)** | **252(85.4%)** |  |
| **Yes** | **190(12.7%)** | **147(12.2%)** | **43(14.6%)** |  |
| **Complications, N (%)** |  |  |  |  |
| **Pulmonary infection** |  |  |  | **0.060** |
| **No** | **1299(86.7%)** | **1053(87.5%)** | **246(88.4%)** |  |
| **Yes** | **199(13.3%)** | **150(12.5%)** | **49(16.6%)** |  |
| **Ventricular arrhythmia** |  |  |  | **0.290** |
| **No** | **1281(85.5%)** | **1023(85.0%)** | **258(87.5%)** |  |
| **Yes** | **217(14.5%)** | **180(15.0%)** | **37(12.5%)** |  |
| **Acute kidney injury** |  |  |  | **0.600** |
| **No** | **1382(92.3%)** | **1112(92.4%)** | **270(91.5%)** |  |
| **Yes** | **116(7.7%)** | **91(7.6%)** | **25(8.5%)** |  |
| **Stress ulcer** |  |  |  | **0.253** |
| **No** | **1493(99.7%)** | **1200(99.8%)** | **293(99.3%)** |  |
| **Yes** | **5(0.3%)** | **3(0.2%)** | **2(0.7%)** |  |
| **Echocardiogram results, mean±SD** |  |  |  |  |
| **LVEDD** | **55.86±5.70** | **55.79±5.59** | **56.12±6.16** | **0.525** |
| **LADD** | **37.17±3.95** | **36.61±3.47** | **39.43±4.86** | **<0.001** |
| **EF** | **51.34±8.15** | **52.22±7.99** | **47.79±7.83** | **<0.001** |
| **CO** | **6.04±2.08** | **6.06±2.09** | **5.97±2.06** | **0.496** |
| **Laboratory data, mean±SD** |  |  |  |  |
| **WBC** | **9.96±1.16** | **9.83±1.08** | **10.53±1.31** | **<0.001** |
| **Hemoglobin** | **129.34±17.91** | **129.69±17.72** | **127.92±18.66** | **0.157** |
| **PLT** | **178.57±58.55** | **178.76±57.81** | **177.79±61.57** | **0.549** |
| **Total cholesterol** | **5.09±1.39** | **5.08±1.37** | **5.16±1.49** | **0.364** |
| **Triglycerides** | **1.55±0.34** | **1.52±0.33** | **1.66±0.36** | **<0.001** |
| **High density lipoprotein** | **1.16±0.18** | **1.17±0.18** | **1.15±0.16** | **0.924** |
| **Low density lipoprotein** | **2.94±0.67** | **2.94±0.65** | **3.10±0.71** | **<0.001** |
| **Glycosylated hemoglobin** | **5.78±1.21** | **5.78±1.18** | **5.74±1.33** | **0.246** |
| **Random Blood Sugar** | **7.75±2.90** | **7.75±2.89** | **7.79±2.93** | **0.136** |
| **hsCRP** | **5.21±2.86** | **4.41±2.08** | **8.49±3.23** | **<0.001** |
| **cTNI** | **3.43±1.75** | **3.40±1.73** | **3.54±1.83** | **0.167** |
| **NT-proBNP** | **1169.17±217.21** | **1125.57±191.43** | **1346.97±225.64** | **<0.001** |
| **Potassium** | **3.89±0.50** | **3.93±0.47** | **3.73±0.58** | **<0.001** |
| **Sodium** | **141.95±5.10** | **141.98±5.08** | **141.80±5.20** | **0.541** |
| **Albumin** | **40.06±6.56** | **40.12±6.37** | **39.45±7.26** | **0.183** |
| **Type of AMI, N (%)** |  |  |  | **0.701** |
| **STEMI** | **731(48.8%)** | **590(49.0%)** | **141(47.8%)** |  |
| **NSTEMI** | **767(51.2%)** | **613(51.0%)** | **154(52.2%)** |  |
| **Killip class, N (%)** |  |  |  |  |
| **Killip ≤ 2** | **1232(82.2%)** | **1004(83.5%)** | **228(77.3%)** | **0.013** |
| **Killip > 2** | **266(17.8%)** | **199(16.5%)** | **67(22.7%)** |  |
| **Culprit lesion** |  |  |  |  |
| **LM, N (%)** | **36(2.403)** | **30(2.5%)** | **6(2.0%)** | **0.644** |
| **LAD, N (%)** | **1042(69.559)** | **827(68.7%)** | **215(72.9%)** | **0.166** |
| **LCX, N (%)** | **1122(74.900)** | **891(74.1%)** | **231(78.3%)** | **0.132** |
| **RCA, N (%)** | **930(62.083)** | **737(61.3%)** | **193(65.4%)** | **0.187** |
| **Values are presented as mean±standard deviation, median (interquartile range), or number (percentage) as appropriate, SD standard deviation, BMI Body mass index, COPD chronic obstructive pulmonary disease, EF ejection fraction, LVEDD left ventricular end-diastolic volume, LADD left atrial diameter, CO cardiac output, WBC white blood cell count, PLT platelet count, CRP C-reactive protein, LM left main coronary artery, LAD left anterior descending artery, LCX left circumflex artery, RCA right coronary artery** | | | | |
